# Primary Stress Factors and Adaptive Mechanisms of Microalgae in Space Environments and Their Applications in Space Life Support Systems

**DOI:** 10.3390/plants15050697

**Published:** 2026-02-26

**Authors:** Siyao Dai, Weiying Feng, Jeffrey Lae, Xuezheng Yu, Chia Min Ho, Fang Yang, Qingfeng Miao, Pengcheng Duan

**Affiliations:** 1 School of Materials Science and Engineering, Beihang University, Beijing 100191, China; 2 J L Algae Science LB, Macgregor, Brisbane, QLD 4109, Australia; 3 State Key Laboratory of Environmental Criteria and Risk Assessment, Chinese Research Academy of Environmental Sciences, Beijing 100012, China; 4 College of Water Conservancy and Civil Engineering, Inner Mongolia Agricultural University, Hohhot 010018, China; 5 Inner Mongolia Algal Life Science Co., Ltd., Ulanqab 011800, China

**Keywords:** microalgae, space environment, deep space exploration, space radiation

## Abstract

Cyanobacteria and eukaryotic microalgae (collectively referred to here as ‘microalgae’) represent early-evolving oxygenic phototrophs and are widely considered ideal candidates for bioregenerative life support systems (BLSS) due to their high metabolic efficiency and ecological robustness. Photosynthetic systems centered on microalgae show strong promise for life support in future deep-space exploration. However, the space environment imposes a range of harsh stressors, including intense radiation, extreme temperature fluctuations, vacuum conditions, and microgravity, all of which critically challenge biological survival. The capacity to resist extreme environments, maintain viability, and reproduce is of great significance. This review systematically summarizes the responses and adaptive mechanisms of microalgae in extraterrestrial settings, including the regulation of radiation defense, photosynthetic metabolic reprogramming, structural protection, and dormancy strategies. Furthermore, the practical applications of microalgae in BLSS encompass atmospheric regulation, food supplementation, and wastewater recycling. By highlighting both achievements and current limitations, this review provides insights into the potential of microalgae as a cornerstone for future long-duration space missions and planetary base construction, while identifying key directions for future research on strain improvement, photobioreactor optimization, and system integration.

## 1. Introduction

As space exploration continues to advance, the development of sustainable life support systems represents an urgent objective. Various countries have conducted research and applied initiatives in this field, including the long-term Environmental Control and Life Support System (ECLSS) aboard the International Space Station (ISS), the “Lunar Palace 1” bioregenerative life support system experiment of China, and the Controlled Ecological Life Support System (CELSS) program of NASA [1,2]. Dating back approximately 2.4–2.1 billion years ago, as primordial oxygenic microbes, ancient cyanobacteria triggered the Great Oxidation Event, which profoundly influenced the evolution of life on Earth [3,4]. Since the Mesoproterozoic (approximately 1.7 billion years ago), marine eukaryotic algae have oxygenated the oceans through photosynthesis and contributed substantially to marine carbon cycling [5]. Having survived five mass extinction events, microalgae have played a pivotal role over evolutionary time [4,6]. Their high photosynthetic efficiency and resilience to extreme environments position them among the leading biological candidates for space life support systems [7]. Therefore, space exposure experiments involving microalgae not only enhance understanding of the boundaries of life but also carry strategic significance for long-term space habitation and deep space missions by informing the optimization of life support and resource utilization technologies.

Numerous countries have conducted flight-relevant trials and simulations on both cyanobacteria and eukaryotic microalgae to evaluate their survival under space conditions (Table 1). Prior studies have demonstrated that specific microalgal species can persist over long durations beyond Earth and exhibit notable adaptability and self-repairing capabilities. For example, in the Biology and Mars Experiment (BIOMEX) of the European Space Agency (ESA), the green alga *Sphaerocystis* sp. retained partial viability after space exposure. Similarly, in the EXPOSE-E mission (an ESA astrobiology mission 1.5 years in space), the desert cyanobacterium *Chroococcidiopsis* sp. CCMEE 029 survived a 548-day exposure period on the exterior of the ISS under a 3 mm sandstone layer [2,8]. These organisms are not only potential components of bioregenerative life support systems (BLSS) capable of producing oxygen and essential nutrients for astronauts but contribute to purifying wastewater and fixing CO_2_, thereby supporting closed-loop ecology [4,9]. In the Micro-ecological life support system alternative (MELiSSA) system, *Chlorella vulgaris* (*C. vulgaris*) fixed approximately 1.4–1.6 kg CO_2_ per kg of dry biomass per day, while releasing an equivalent amount of O_2_ [10]. In such systems, microalgae can effectively regulate oxygen and carbon dioxide concentrations to maintain atmospheric balance and use human waste (e.g., urine) as a growth substrate [9]. These attributes provide both theoretical support and practical directions for the use of microalgae in off-world colonization and the development of space-based life support systems.

Despite these promising findings, robust experimental data on microalgal exposure to space remain limited [17]. Moreover, comprehensive studies addressing the impact of unique space environmental factors, such as high radiation, extreme temperatures, vacuum, and microgravity, on microalgal growth remain inadequate. In light of recent advances, this review synthesizes the current findings on the key stressors in extraterrestrial settings and the corresponding adaptive mechanisms in microalgae, aiming to inform future space biology experimental designs and technological developments. Additionally, it examines the practical applications of microalgae in life support systems, with particular attention paid to oxygen production, food supplementation, and wastewater treatment, while delineating the key challenges and research directions for their deployment in long-duration space missions.

## 2. Primary Stress Factors of Space Environments on Microalgae

**Figure 1:**
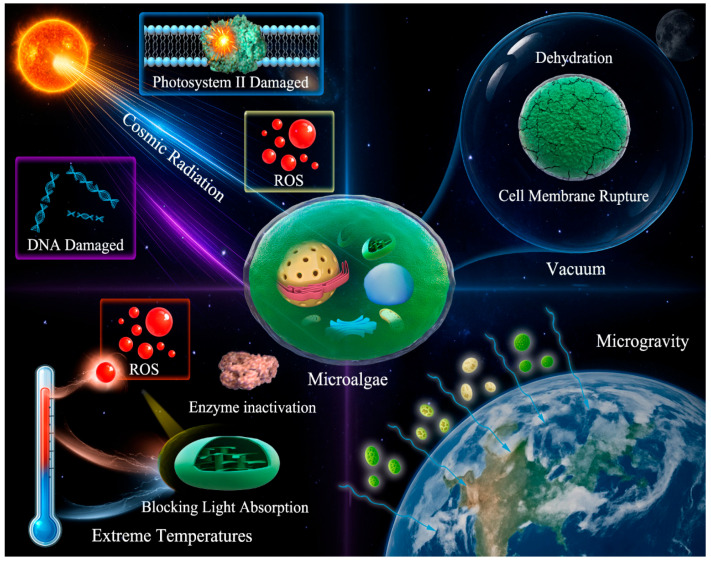
Influence factors of the space environment on microalgae.

As shown in Figure 1, the space environment exerts diverse pressures, with cosmic rays being major stressors. Microalgae must cope with the combined effects of diverse types of radiation. Although multiple radiation threats reduce photosynthetic efficiency, they offer a unique opportunity to study defense pathways. Long-term in-orbit experiments are needed to elucidate the response patterns of microalgae to real-space radiation environments. Vacuum exposure is another significant stressor that can lead to cell dehydration, membrane structure rupture, and other issues that threaten the physiological integrity of microalgae and can cause cumulative metabolic damage. Extreme temperature fluctuations in space pose additional challenges. Both high and low temperatures inhibit photosynthesis, and abrupt and extreme temperature swings can severely damage cellular structures. In a microgravity environment, most microalgae show high resilience; however, their responses are species-specific. The impact of microgravity on microalgae depends on factors such as species, size, and cell structure; however, the underlying mechanisms remain unclear and warrant further investigation.

### 2.1. Influence of Space Radiation on Microalgae

Microalgal oxygenic photosynthesis is primarily driven by photosynthetically active radiation (400–700 nm), whereas ultraviolet radiation is generally treated as a stressor due to its higher photon energy and propensity to induce photodamage and oxidative stress, particularly in the UVB (mid-wave ultraviolet) and UVC (short-wave ultraviolet) ranges [10,18,19,20]. Although some ground-based studies have suggested that low-dose radiation may enhance microalgal performance by enhancing photosynthesis or triggering metabolic adjustments, these findings are typically based on simplified models involving single radiation types and short-term low-intensity exposure [17,21]. Beyond Earth, microalgae are exposed to a combination of ultraviolet radiation, Solar Energetic Particle (SEP) events, galactic cosmic rays (GCR), and secondary neutron radiation [15,22]. Space exposure experiments indicate that high-energy particles from solar activity and supernovae, in conjunction with ultraviolet radiation of solar origin, can directly damage microalgal DNA (e.g., by forming pyrimidine dimers) and degrade photosynthetic pigments, such as chlorophyll and phycobiliproteins, leading to impaired photosystem II (PSII) function [11,15,23,24]. Radiation-induced oxidative stress increases levels, which attack lipids, proteins, and DNA, and trigger apoptosis or mutations [2,11,17]. The high-energy proton flux during SEP events induces intense ionization, directly compromising DNA integrity and disrupting cellular metabolism [22,25]. Neutron radiation, arising secondarily from cosmic rays interacting with spacecraft materials, is highly penetrating and may induce mutations in microalgae [26]. Although these multiple radiation threats compromise photosynthetic efficiency, viability, and genetic stability, they also enable investigation of the resilience mechanisms of microalgae. Given the complex, multi-source, high-energy, and chronic nature of space radiation, prolonged in-orbit studies are essential to fully understand the actual responses of microalgae to space radiation environments.

### 2.2. Influence of Extreme Vacuum Conditions on Microalgae

Vacuum exposure is a further major environmental stressor for microalgae. Low-pressure or vacuum conditions induce cellular dehydration and membrane rupture [11,27,28]. Membrane damage allows endogenous enzymes to access pigments, proteins, and lipids, promoting their degradation and resulting in nutrient depletion. Because many enzyme activities are temperature-dependent, evidence indicates that degradation under combined vacuum and high temperature (e.g., 75 °C) is more severe than under either stress alone [27]. Although some microalgal species, such as *Chroococcidiopsis* sp., *Nostoc* sp., and *C. vulgaris*, demonstrate short-term vacuum tolerance and the potential for recovery, long-term exposure substantially compromises their physiological activity [2,15]. In the BIOMEX, desiccated microalgae were exposed outside the ISS under simulated Martian and space conditions; after seven days of vacuum exposure, DNA amplification failed, although nuclear structures remained [2]. Ground-based vacuum-only studies have also revealed that *Arthrospira platensis* exhibits significant losses of pigments, lipids, and phycocyanins under vacuum conditions. These results suggest that vacuum exposure threatens the physiological integrity of microalgae and can lead to cumulative metabolic damage.

### 2.3. Influence of Extreme Temperatures on Microalgae

Extreme temperature fluctuations in space pose additional challenges for microalgal survival. Both high and low temperatures substantially diminish photosynthetic activity [17]. At low temperatures, cells alter their photochemical processes to curb light absorption and protect themselves from excitation-induced stress. Cold conditions also diminish carboxylase function, thereby impairing the efficient use of absorbed photons for carbon fixation. In contrast, high temperatures inhibit PSII charge separation and oxygen evolution, thereby increasing reactive oxygen species (ROS), which damage cellular biomolecules [10,17]. In realistic space settings, abrupt and large-amplitude temperature swings can severely disrupt cellular structure, requiring microalgae to exhibit broad-spectrum thermal resilience. Wang et al. exposed desert *Chlorella* to stratospheric Mars-analog conditions, finding that temperature variations from −19 °C to 71 °C caused membrane and protein denaturation as well as mitochondrial swelling [15]. In the BIOMEX, freeze–thaw cycles (−10 °C/+45 °C) and high-temperature exposure (−25 °C and 60 °C) caused minor DNA damage but may suppress metabolism over longer durations [11]. Although many microalgae from the extreme environments of Earth are thermotolerant, repeated freeze–thaw cycles nonetheless elevate membrane rupture rates and reduce cellular activity.

### 2.4. Influence of Microgravity on Microalgae in the Extreme Space Environment

Plants commonly exhibit atypical growth patterns under microgravity. However, many microalgae demonstrate survivability, but their physiology can change significantly, and this is not always adaptation in a positive sense, but sometimes a stress response [10,29]. Microgravity can reduce the rates of convection and diffusion of nutrients and metabolites around cells and suppress sedimentation and aggregation, thereby influencing physiological responses [7,10,30]. Nevertheless, studies indicate that microalgal responses to microgravity are not universal across species [7,31]. Particular species such as *C. vulgaris* exhibit robust growth and adaptability under simulated microgravity, whereas others can experience metabolic disturbances or growth inhibition [17,32]. In 1968, the USSR conducted early experiments with *Chlorella* aboard the Salyut space station, which indicated that microgravity does not interfere with algal growth [31]. Aboard the ISS, the *Arthrospira*-B experiment achieved the first remote real-time monitoring of oxygen production and growth rate in *Arthrospira* (Spirulina), showing no significant reduction in oxygen yield under microgravity [7,31]. Multiple Artificial-Gravity Research System (MARS) in Japan simulated Martian gravity (0.38 g) on the ISS and found no significant differences in microalgal growth compared to conditions on Earth [31]. However, *Microcystis aeruginosa* exhibits growth inhibition in microgravity [7]. The effect of microgravity on microalgae is governed by the species, size, and cellular structure [33,34]. Nevertheless, the underlying mechanism remains unclear and warrants study.

## 3. Adaptive Mechanisms of Microalgae in the Extreme Space Environment

**Figure 2:**
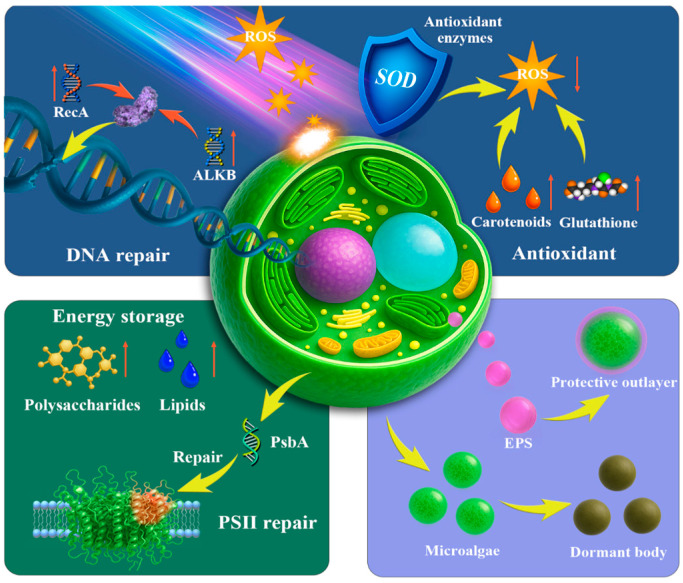
Diagram of the adaptive mechanism of microalgae in space environment.

During the BIOMEX mission on the ISS, *Chroococcidiopsis* sp. was exposed to space conditions for 1.5 years. Following retrieval and rehydration, cyanobacteria resumed growth, demonstrating remarkable tolerance to long-term exposure in near-planet orbital space [2]. Similarly, *Chlorella* sp. cells were exposed to near-space conditions in the stratosphere for three hours, experiencing ultraviolet irradiation, low-pressure exposure, and thermal oscillations. After recovery, a fraction of the population remained viable, indicating partial resistance to extreme short-term exposures [2,31]. Together, these cases span two major microalgal groups, cyanobacteria and eukaryotic microalgae, which can exhibit both shared and distinct stress response systems under extreme space environment. These survival outcomes suggest that microalgae activate layered defense networks in harsh environments (Figure 2). Understanding these processes is essential for harnessing the biotechnological potential of microalgae for space exploration and improving applied strategies.

### 3.1. Antioxidant Defense and DNA Repair

Many cyanobacteria are extremophiles with exceptionally robust antioxidant and DNA repair systems. *Chroococcidiopsis* sp. endures near-space Mars-like conditions with high survival, as it can survive years in a dried state and withstand up to 13 kJ/m^2^ of UVC radiation and 12 kGy of gamma radiation [35]. Such resilience is attributed to (i) protective pigments (e.g., UV-screening scytonemin and carotenoids) that quench ROS; (ii) ROS-scavenging enzymes (e.g., superoxide dismutase, catalase); and (iii) efficient repair of UV- and radiation-induced DNA damage (photolyase, excision repair) [35,36,37,38]. *Nostoc* sp. exposed to Mars-like radiation activates antioxidant metabolic pathways, particularly the ascorbate–glutathione cycle, and upregulates the catalase-encoding gene *katB* to enhance reactive oxygen species scavenging. *Nostoc* sp. also upregulates ribose and phosphate transport genes (e.g., *rbsC*, *pstA*, *pstC* and *pstS*), supporting nucleotide precursor supply for repairing UV-induced DNA damage [39]. Cyanobacteria often maintain multiple genome copies and DNA-binding proteins, allowing rapid post-damage genome reconstitution [35,40,41]. This arsenal of antioxidant and DNA repair mechanisms enables cyanobacteria to remain viable after space exposure, with only minimal mutations due to their robust DNA repair genes [42].

The antioxidant defenses and DNA repair mechanisms activated by eukaryotic microalgae under extreme conditions are broadly similar to those of cyanobacteria, including the ascorbate–glutathione cycle, Rad51-mediated homologous recombination in DNA damage repair, and ROS scavenging by antioxidant pigments such as β-carotene and astaxanthin [15,23,31,43]. However, ISS-related experiments have shown that the unicellular green alga *Chlamydomonas reinhardtii* can accumulate more DNA mutations under microgravity than ground controls (approximately a 50% increase), highlighting a marked contrast with the genetic stability often observed in cyanobacteria [44].

### 3.2. Photosynthetic Adjustment and Energy Storage

Under space-induced stress, microalgae reprogram their photosynthetic and respiratory pathways to accumulate energy-rich compounds and enhance stress resilience (Figure 2). Microalgae can enhance PSII repair capacity by accelerating D1 (PsbA) turnover and the PSII repair cycle under photodamage stress [28,45]. Concomitantly, microalgae remodel the supramolecular organization of thylakoid membrane complexes, which can mitigate light-induced damage and help maintain light-harvesting function [46,47]. To sustain carbon assimilation, microalgae regulate CO_2_ concentrating mechanism (CCM) components (e.g., carboxysomes in cyanobacteria and pyrenoid-based CCM in green algae), thereby supporting RuBisCO performance and CO_2_ fixation [48,49]. In eukaryotic microalgae, lipid accumulation increases under radiation or nutrient stress, providing an energy reserve that supports subsequent cellular demands [12,45,50]. Lipids not only act as carbon reserves but also exhibit radical-quenching capacity while stabilizing bilayers [12]. After exposure to space or near-space stressors, reduced growth or a prolonged lag phase during recovery may indicate a transient metabolic slowdown that reallocates resources toward cellular repair [15].

### 3.3. Structural Protection and Dormancy Defense

Microalgae can increase tolerance in hostile settings through structural adaptations such as extracellular polymer secretion (EPS) and the formation of dormant structures and heterocysts [17,45,51]. Cellular structure is also central in environmental resilience (Figure 2). Cyanobacteria, such as *Nostoc*, can secrete EPS and generate shielding envelopes under desiccation, vacuum, or radiation stress to stabilize the cell wall, reduce water loss, and improve survival in space [2,18,31]. Some algae form dormant structures, such as akinetes in cyanobacteria and hypnospores in green algae, and cyanobacteria can also differentiate into heterocysts. These specialized structures contribute to resilience over extended durations under nutrient-deficient or resource-limited conditions [31]. Space-tolerant cyanobacteria, such as *Chroococcidiopsis*, possess robust microstructures that offer superior protection against ambient extremes [12]. Cyanobacteria also have an intriguing ability to reduce UVR toxicity by employing UV-absorbing or screening substances, such as mycosporine-like amino acids and scytonemin [38]. Experimental evidence suggests that microalgal cells smaller than 10 μm, owing to their compact size and structural uniformity, acclimate more readily to microgravity [7]. Cells with more rigid cytoskeletal structures also exhibit greater resistance to gravitational stress than those with more flexible structures [7].

## 4. Application of Algae in Space Life Support Systems

**Figure 3:**
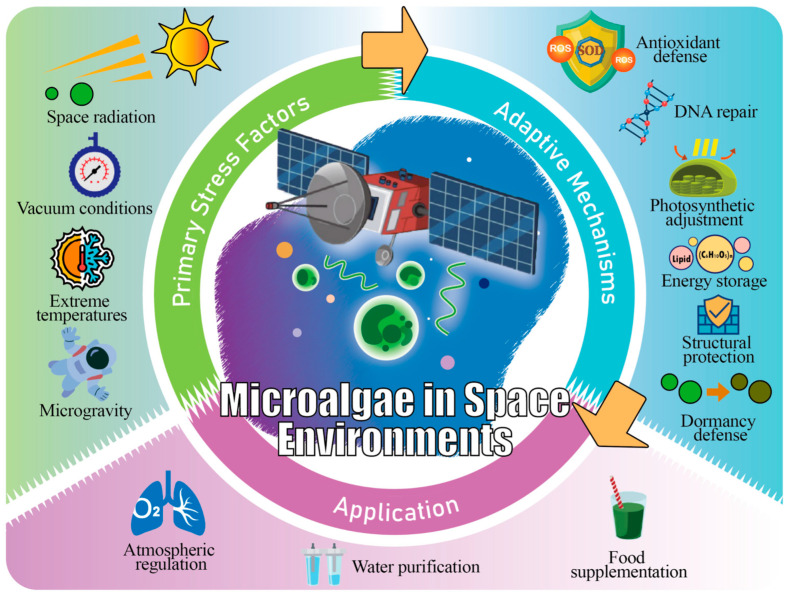
Application potential driven by microalgal adaptive responses to space stress.

These physiological and structural adaptations of microalgae in hostile extraterrestrial settings not only ensure their survival under conditions such as vacuum, radiation, extreme temperatures, and microgravity but also lay a solid foundation for their practical application in life support systems. Therefore, microalgae are considered key components for the construction of closed-loop BLSS. As shown in Figure 3, they can sustain proliferation and deliver multiple roles, including atmospheric regulation, food and nutrient supply, water purification, and waste recycling, demonstrating significant advantages in long-term deep-space exploration and planetary base construction [52,53].

### 4.1. Atmospheric Regulation

Current spacecraft primarily rely on physicochemical oxygen supply methods such as water electrolysis for O_2_ production and solid oxygen candles [54,55]. These systems function as partly closed life support methods that depend on external supplies and resource reserves. If the mission duration is excessively long or supplies are limited, sustainability becomes challenging. Microalgae convert carbon dioxide into oxygen through photosynthesis, a process that can theoretically operate indefinitely, providing improved persistence and closed-loop performance and serving as an essential supplement to physicochemical methods [53]. Microalgae possess a CCM, facilitating effective CO_2_ uptake under dilute conditions and dynamically regulating oxygen production rates to maintain gas balance in closed environments [56]. *Chlorella* sp. can achieve a peak oxygen production rate of 15 mmol/(L·h). Assuming 0.84 kg/d per person, approximately 73 L of *Chlorella* culture can meet the daily oxygen demand of one astronaut [56]. In addition to meeting human respiratory needs, algae provide oxygen to other fauna within closed biomes. Experiments have shown that an 11 L *Euglena* culture system can operate for over 600 days absent resupply and sustain the respiratory needs of four fish and 15 snails through photosynthesis [57]. These studies imply that algae can not only serve as oxygen production units but also contribute to building stable closed ecosystems. Compared to single physicochemical methods, microalgae enable prospects of partial or complete regenerative life support.

### 4.2. Food Supplementation

In space missions, restricted volume, scarce supplies, and long-term enclosed environments impose high demands on astronaut nutritional supplies. Owing to their renewability and multifunctionality, algae are progressively viewed as prime options for space food [10]. Table 2 summarizes the applications and nutritional content of microalgae employed in food products. Algae can serve as a high-quality protein source, with protein content accounting for 50–70% of the dry weight and a complete amino acid profile that meets human essential amino acid requirements [57,58]. Algae are rich in polyunsaturated fatty acids, particularly ω-3 fatty acids such as eicosapentaenoic acid (EPA) and docosahexaenoic acid (DHA), that support cardiovascular and neurological health in astronauts under microgravity conditions [59]. Algae also contain abundant vitamins, minerals, and dietary fibers, helping to offset nutritional deficiencies in prepackaged space foods and preventing anemia, osteoporosis, and metabolic disorders [59,60,61]. Additionally, pigments (e.g., phycocyanin and carotenoids) and polysaccharides in algae exhibit strong antioxidative and immune-modulating actions, protecting astronauts from oxidative damage caused by radiation and stress in space, thereby reducing the risk of chronic diseases and inflammation [59,60]. However, not all algal taxa are suitable for human consumption. For example, some green algae may cause digestive discomfort even at small doses, although they still remain useful as feed for fish and other animals [57]. Furthermore, most algae are carbohydrate-poor; for instance, *Spirulina platensis* has a carbohydrate proportion of only 15–25%, making it difficult to use as a primary energy source or staple food [57]. In summary, algae are suitable as auxiliary mixed options in conjunction with conventional staple food sources for enhancing the overall nutritional security of astronauts and other organisms during long-term space missions.

### 4.3. Water Purification

Algae can assimilate nutrients such as nitrogen, phosphorus, and chemical oxygen demand (COD) from human metabolic waste (e.g., urine) and domestic wastewater, serving biological purification and water recycling functions [52,77,78]. Evidence indicates that microalgal species such as *Chlorella* and *Scenedesmus* exhibit high nitrogen and phosphorus removal rates in appropriately diluted urine or wastewater treatment experiments, converting wastewater into resources that can be further purified [10,79]. Relative to conventional onboard treatment technologies (e.g., physical filtration, distillation, chemical oxidation, and ion exchange), algal systems not only remove pollutants but also assimilate elements such as nitrogen and phosphorus, converting them into usable biomass. Microalgae cultivated in wastewater often build substantial lipid–protein stores, enabling resource recovery, reducing external material supply demands, and enhancing the closed-loop efficiency of the system [78,79]. Currently, laboratory cultivation of algae routinely uses photobioreactors (PBRs), such as biofilm reactors, which have high algal density and stability to achieve COD, nitrate–nitrogen, and total phosphorus removal rates of 87%, 91%, and 93%, respectively, offering significant advantages in treatment efficiency and biomass recovery [80,81]. Therefore, integrating algae into the water recycling segment of space life support systems offers the potential to enhance waste resource utilization while improving the closed-loop efficiency and long-term operational reliability of the system.

## 5. Research Prospects and Challenges

Future research must be aligned with mission requirements for beyond-Earth operations, such as oxygen production, ecological recycling, food supplementation, and extraterrestrial habitability assessments. In algal PBRs designed for life support applications, illumination is a key limiting factor affecting microalgal growth rates and oxygen production efficiency [82]. For deep-space missions, both the intensity and spectral composition of natural light vary with orbital conditions, making stable and precise control of irradiance difficult [83]. Therefore, a more practical engineering approach is to harvest solar energy, store it in batteries, and then power LEDs with appropriate spectral and optical characteristics to provide controllable lighting for both PBR and greenhouse systems [82]. Importantly, lighting is often the largest electrical power demand in PBR operation, thereby constraining scalable oxygen production. Meanwhile, nearly all electrical energy consumed by LEDs ultimately enters the spacecraft’s thermal balance as heat, so heat rejection capacity constitutes an equally important engineering challenge [10,82,84]. The development of highly efficient and energy-saving LED illumination systems not only enhances photon economy but also reduces overall energy consumption, thereby improving the sustainability of cultivation systems under limited energy conditions [10,84]. From a biological perspective, selecting highly stress-resistant strains and employing genetic engineering to optimize photosynthetic genes could boost O_2_ output while significantly enhancing the adaptability of microalgae to extreme space environments, such as radiation and microgravity [31]. Regarding food applications, priorities include algal breeding programs aimed at increasing the nutritional quality, improving palatability, and enhancing the bioavailability of essential nutrients to better meet the dietary needs of astronauts for long-term missions [10,56,57]. Additionally, advances in large-scale and automated harvesting and processing technologies are crucial for the stable, efficient, and sustainable application of microalgae in aerospace engineering. Evaluating the capacity of microalgae to utilize in situ resources (ISRU) in regolith and lithic matrices helps assess the feasibility of localized algal cultivation, offering experimental models for planetary base development and assessing planetary habitability [10,11].

Despite the considerable promise of microalgae in space life support systems, current research faces numerous challenges. First, existing space exposure experiments are limited in both variety and duration, with most studies focusing on short-term exposures and lacking the long-duration in-orbit data essential for understanding the true adaptation over extended expeditions [2,15]. Moreover, most studies have primarily examined combined impacts of space stressors (e.g., radiation and vacuum), offering limited insights into specific response mechanisms. Owing to the difficulties in fully simulating space conditions on Earth and the logistical constraints of long-term space experiments, systematic investigations into the independent and synergistic effects of environmental variables are uncommon [10]. Species-specific variability also poses hurdles; different microalgae exhibit distinct responses to space environments, and the lack of robust comparative studies limits the accurate identification of the most resilient strains. The experimental data obtained from microalgal exposure studies can shape the architecture of microalgal growth systems. At present, technical obstacles also persist in developing space-based microalgal growth systems, particularly concerning energy consumption, system stability, and contamination control, while also taking into account economic costs and construction complexity [1,10,17]. To maintain an axenic environment, microalgal inoculation requires the bioreactor to be equipped with sterilizable inoculation ports and compatible connection systems. During cultivation, routine monitoring and periodic removal of dead cells are also necessary, making long-term maintenance costly [32,85]. Moving forward, research must shift beyond molecular-level mechanistic studies to system-level optimization of bioreactors to address the key technical barriers to practical applications [17].

## 6. Conclusions

Microalgae exhibit multilayered responses and adaptive mechanisms in hostile extraterrestrial settings, highlighting their immense potential as core biological resources for future deep-space life support systems. Key space stressors, including high radiation, vacuum, extreme temperatures, and microgravity, pose substantial hazards to algal growth. Nevertheless, particular microalgal species maintain their viability and resume growth in extreme extraterrestrial conditions by activating antioxidant defenses and DNA repair systems, reconfiguring their photosynthetic metabolism, and developing physical protective structures. These adaptive traits enable the microalgae to support essential roles in closed-loop ecosystems, including oxygen generation, carbon fixation, waste recycling, and nutrient cycling. They deliver key capabilities for gas regulation, food supplementation, and water purification, underscoring their strategic value in sustaining long-duration human space missions.

However, the suitability of microalgae for specific tasks within a Bioregenerative Life Support System (BLSS) is highly species-dependent and closely linked to their metabolic and structural traits. Based on comparative analysis of space exposure experiments, cyanobacteria—particularly those capable of forming multilayered biofilms, such as *Chroococcidiopsis* sp.—demonstrate superior resistance to the complex and combined stressors of open space, including vacuum, UV radiation, and temperature extremes. Their robust extracellular polymeric substances (EPS), efficient DNA repair mechanisms, and ability to enter dormant states make them ideal candidates for long-term exposure and in situ resource utilization (ISRU) on planetary surfaces.

Further evidence from Mars regolith simulant studies reinforces this view: *Nostoc muscorum* showed stable growth and sustained photosynthetic efficiency, while Anabaena cylindrica exhibited browning and metabolic decline, and Chlorella vulgaris failed to grow altogether. These contrasting responses underscore the metabolic flexibility and ecological resilience of certain cyanobacteria over eukaryotic microalgae in resource-limited and extreme environments. Thus, cyanobacteria such as *Chroococcidiopsis* and *Nostoc* emerge as the most promising primary producers for ISRU-based life support systems, owing to their physical robustness, genetic stability, and ability to function under multistress conditions.

Understanding these species-specific adaptive mechanisms is not only of biological interest but also directly informs the design of next-generation engineering solutions. For instance, the biofilm-forming capacity and EPS production observed in resilient cyanobacteria can inspire the development of advanced photobioreactors that mimic protective biofilm architectures, enhancing cell survival and system stability. Likewise, insights into DNA repair pathways and antioxidant defense networks can guide genetic engineering efforts to enhance stress tolerance in less robust but nutritionally valuable strains. By translating biological knowledge into engineering principles—such as optimizing light delivery, automating harvesting, and integrating contamination control—we can move toward scalable, reliable, and sustainable BLSS for long-duration space missions.

In conclusion, the integration of mechanistic understanding with applied system design is essential to fully unlock the potential of microalgae for space exploration. Future research should prioritize long-term in-orbit validation, strain improvement through synthetic biology, and the development of hybrid systems that combine biological and physicochemical processes. Such efforts will not only advance the feasibility of human habitation beyond Earth but also deepen our understanding of life’s resilience in extreme environments.

## Figures and Tables

**Table 1:** Habitat and survival status of algae under extreme conditions in space environment.

**Algal Species**	Sample Treatment	Habitat Environment	Survival Situation	References
Cyanobacterium *Chroococcidiopsis* sp. CCMEE 029	Dried cells mixed with S-MRS Mars analog minerals; partially shielded with MgF2 filters simulating subsurface conditions	Simulated Mars-like conditions, low Earth orbit	Survived and recovered well, low DNA damage especially in shielded areas	[2]
Cyanobacterium *Chroococcidiopsis* sp. CCMEE 029	Cells mixed with Mars regolith analogue (S-MRS), dried	Dried biofilms and planktonic cells exposed to simulated space vacuum, UVC, Martian atmosphere, and thermal cycling	Biofilms survived better than planktonic cells, retained DNA and pigments under high UVC	[11]
*Anabaena cylindrica* PCC 7122	Grown in aqueous extract of MGS-1 (Mars regolith simulant)	Mars regolith extract (MGS-1), Earth-like atmosphere, low nutrients	Photosynthetic efficiency dropped after day 15; biomass turned brownish by 3rd week	[12]
*Nostoc muscorum* UTAD_N213	Grown in aqueous extract of MGS-1 (Mars regolith simulant)	Mars regolith extract (MGS-1), Earth-like atmosphere, low nutrients	The survival condition was favorable; stable growth and PSII efficiency > 0.40	[12]
*Arthrospira platensis* UTEX LB 2340	Grown in aqueous extract of MGS-1 (Mars regolith simulant)	Mars regolith extract (MGS-1), Earth-like atmosphere, low nutrients	Survived; mild or no quantifiable growth; fluorescence signal unreliable due to clogging	[12]
*Nostoc punctiforme*	Filtered onto cellulose acetate membrane and dried	Stored at ambient temperature on the ISS for 34 days	The microalgal cells died, but the carried plasmids remained intact	[13]
*Limnospira indica* PCC 8005	Cultured in a 50 mL membrane photobioreactor on the ISS	Algae cultivation chamber in the MELiSSA closed-loop system	Achieved CO_2_ fixation, O_2_ production, and primary food synthesis, but nutrient recovery from waste remained challenging	[14]
*Chlorella vulgaris*	Grown in aqueous extract of MGS-1 (Mars regolith simulant)	Mars regolith extract (MGS-1), Earth-like atmosphere, low nutrients	No growth observed; only thrived in diluted synthetic medium	[12]
*Chlorella* sp. (*Tengger* Desert isolate)	Drying treatment; exposure to ultraviolet light through a quartz glass window	Near space (~31 km altitude), exposed on high-altitude balloon for ~3 h	Most cells survived; reduced photosynthesis; increased mortality; damaged chloroplasts/mitochondria; 3292 differentially expressed genes (DEGs) were detected.	[15]
*Chlamydomonas reinhardtii*	The *Dsup* gene from tardigrades was inserted into the nucleus of *Chlamydomonas reinhardtii*, which was then inoculated onto agar plates	Exposed to deep space radiation during the Artemis-1 lunar flyby mission under 6 h of daily red and blue light	Survival rate was 46.9%; after returning to Earth, all algal cultures exhibited vigorous growth	[16]

**Table 2:** Applications of microalgae in the food field.

Microalga	Applications	Functions (Health Benefits)	Key Nutritional Composition	References
*Spirulina*	Beverages, biscuits, dairy, bread, pasta, snacks	Antioxidant; regulates blood glucose/lipids; immune and probiotic support	Protein: ~60–70% DW; rich in essential amino acids, vitamins, minerals	[60,62,63,64]
*Chlorella*	Health supplements, bakery, meat analogues, beverages	Antioxidant; immune-enhancing; hypoglycemic; hypolipidemic	Protein: 50–60% DW; vitamins; pigments; lipids: up to 30% DW	[64,65,66,67]
*Haematococcus*	Supplements, beverages, vegan gels	Potent antioxidant; anti-inflammatory; supports eye and immune health	Astaxanthin: 3–5% DW (primarily as esters)	[68,69]
*Dunaliella*	Natural pigments, dietary supplements, feed	Antioxidant; immune regulation; eye protection	β-carotene: 10–13% DW (high in 9-cis isomer); ω-3 PUFAs	[70,71,72,73]
*Nannochloropsis*	Food ingredients, fortified dairy, EPA oil	Anti-inflammatory; antioxidant; supports cardiovascular health	Lipids: 20–60% DW; EPA: 20–30% of total fatty acids	[74,75]
*Isochrysis*	Fortified yogurt, fish products, additives	Antioxidant; antibacterial; supports cardiovascular and neural health	Rich in ω-3 PUFAs (DHA and EPA); fucoxanthin	[73,76]

## Data Availability

No data was used for the research described in the article.
